# Nanostructured Metal Oxide from Metallic Glass for Water Splitting: Effect of Hydrothermal Duration on Structure and Performance

**DOI:** 10.3390/ma18174082

**Published:** 2025-08-31

**Authors:** Hae Jin Park, Tae Kyung Kim, Jürgen Eckert, Sung Hwan Hong, Ki Buem Kim

**Affiliations:** 1 Department of Nanotechnology and Advanced Materials Engineering, Sejong University, 209, Neungdong-ro, Gwangjin-gu, Seoul 05006, Republic of Korea; haejinp@sejong.ac.kr (H.J.P.); xorud7829@naver.com (T.K.K.); shhong@sejong.ac.kr (S.H.H.); 2 Erich Schmid Institute of Materials Science, Austrian Academy of Sciences, Jahnstraße 12, 8700 Leoben, Austria; juergen.eckert@oeaw.ac.at; 3 Department of Materials Science Montanuniversität Leoben, Jahnstraße 12, 8700 Leoben, Austria

**Keywords:** metallic glasses, oxide, hydrothermal process, water splitting

## Abstract

This study investigates the optimal duration for forming a uniform oxide layer and evaluates its influence on water-splitting performance. We selected a Ti_50_Cu_32_Ni_15_Sn_3_ amorphous ribbon, which is known to simultaneously form anatase TiO_2_ and Sn oxide via a single hydrothermal process. Hydrothermal treatments were conducted at 220 °C in 150 mL of distilled water for durations of 3 and 6 h. The process successfully formed nanoscale metal oxides on the alloy surface, with the uniformity of the oxide layer increasing over time. The amorphous phase of the alloy was retained under all conditions. X-ray photoelectron spectroscopy (XPS) analysis confirmed the formation of TiO_2_ and SnO_x_, while Cu and Ni remained in their metallic state. Furthermore, we verified the coexistence of these oxides with metallic Ti and Sn. Photoelectrochemical analysis showed that the sample treated for 6 h exhibited the best water-splitting performance, which correlated directly with the most uniform oxide coverage. This time-controlled hydrothermal oxidation method, using only water, presents a promising and efficient approach for developing functional surfaces for electronic and photoelectrochemical applications of metallic glasses (MGs).

## 1. Introduction

Metallic glasses (MGs), which possess a disordered atomic structure, exhibit outstanding properties such as high strength, large elastic limits, and superior corrosion resistance compared to their crystalline counterparts [1,2,3,4]. The combination of these attributes, including their excellent formability, high electrical conductivity, and corrosion resistance, has led to their application in various fields, such as structural, energy, electronic, and electrochemical engineering [5,6,7,8,9,10,11,12,13]. The exceptional corrosion resistance of MGs [14,15,16,17] is particularly advantageous in the harsh, acidic environments typical of water splitting, offering a pathway to overcome the long-term stability issues faced by conventional photoelectrode materials.

A recent study reported a method where a metallic glass ribbon, without atomic-scale phase separation, serves as a precursor for the in situ formation of multifunctional oxides via a hydrothermal process, enabling its use as a photoelectrode [18]. Unlike conventional photoelectrode fabrication, which often involves depositing metal oxides onto conductive glass, this single-step oxidation process offers a compelling advantage: it simplifies manufacturing while expanding the application potential of MGs.

In this study, we investigated a Ti_50_Cu_32_Ni_15_Sn_3_ alloy, previously shown to form both anatase TiO_2_ and Sn oxide on an amorphous ribbon through a single hydrothermal process [18]. The prior study successfully demonstrated the formation of a mixed-oxide nanostructure through a long-duration hydrothermal process of 96 h. In contrast, our work systematically investigates whether a significantly reduced processing time of 3 and 6 h can achieve a similar nanostructure and comparable high photoelectrochemical performance. This approach is critical for developing a more efficient and scalable fabrication method for metallic glass-based photoelectrodes.

A key aspect of obtaining the desired heterostructure, which consists of photo-reactive oxides for water splitting via a simple process, is strategic alloy design and careful selection of constituent elements based on their affinity for oxygen. In this context, we employed a multi-component metallic glass in which atoms are randomly distributed, thereby facilitating the uniform and simultaneous growth of oxides. This structural feature also provides advantages for developing flexible photoelectrodes. For these reasons, the quaternary Ti-Cu-Ni-Sn metallic glass was specifically designed for the water-splitting photoelectrode, with each element chosen according to its unique role in promoting oxide formation and enhancing performance.

Accordingly, this work focused on elucidating how the degree of oxide formation, as a function of hydrothermal processing time, influences the water-splitting ability of the resulting photoelectrodes. The study was conducted to confirm whether multifunctional oxides could be successfully formed on the substrate surface despite the short processing time and to evaluate the impact of oxide growth kinetics on the overall photoelectrochemical performance.

## 2. Materials and Methods

The master alloy of Ti_50_Cu_32_Ni_15_Sn_3_ was strategically designed and prepared for its intended use as a photoelectrode material for water splitting. This quaternary alloy was composed of elements selected based on their specific functional roles and oxygen affinities. Ti and Sn were selected to form a functional oxide with the capability for water splitting, while Cu and Ni were selected for their oxidation resistance to fulfill the necessary mechanical properties and stability of the photoelectrode.

The master alloy Ti_50_Cu_32_Ni_15_Sn_3_ was prepared by arc-melting high-purity elements (>99.99 wt%) under a high-purity argon atmosphere (99.9999%). The resulting ingot was re-melted by induction in a quartz tube under the same atmosphere and then melt-spun onto a rotating copper wheel (surface velocity: 35 m·s^−1^) to produce amorphous ribbons approximately 50 μm thick and 10 mm wide.

Hydrothermal oxidation was performed in 150 mL of distilled water at 220 °C for either 3 h (3HRS) or 6 h (6HRS). Phase identification and structural analysis were conducted using X-ray diffraction (XRD; PANalytical Empyrean, Almelo, Netherlands) with Cu Kα_1_ radiation (λ = 1.5406 Å). Differential scanning calorimetry (DSC) was performed at a heating rate of 20 K·min^−1^ under a high-purity Ar atmosphere. Surface morphology and oxide coverage were examined using field-emission scanning electron microscopy (FE-SEM; Hitachi SU-8010, Tokyo, Japan). XPS depth profiling was performed on the 6HRS samples using a PHI 5000 VersaProbe (Ulvac-PHI, Chigasaki, Japan) spectrometer with monochromatic Al Kα radiation. For the depth profile, the surface was etched with 2 keV Ar+ ions at a calibrated sputter rate of 15 nm/min using a SiO_2_ standard. The binding energies were calibrated by referencing the C 1s peak to 284.6 eV. Spectral analysis and quantification were carried out using MultiPak 9.0 software.

Photoelectrochemical (PEC) measurements were performed in a three-electrode cell. For the working electrodes, the as-spun, 3HRS, and 6HRS ribbons were mounted onto fluorine-doped tin oxide (FTO) glass using a silver paste to ensure robust ohmic contact. A platinum (Pt) mesh was used as the counter electrode, and an Ag/AgCl electrode as the reference electrode. Linear sweep voltammetry (LSV) and chronoamperometry (CA) were conducted in 1 M HClO_4_ (pH 0) under both dark and AM 1.5G illumination (100 mW·cm^−2^) conditions. All measured potentials were converted to the reversible hydrogen electrode (RHE) scale using the following equation:

E_RHE_ = E_Ag/AgCl_ + E^0^_Ag/AgCl_ + 0.059 × pH. (1)

## 3. Results and Discussion

**Figure 1:**
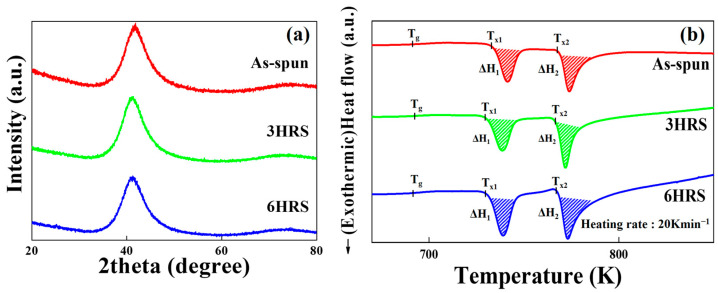
Structural analysis of the Ti_50_Cu_32_Ni_15_Sn_3_ alloy. (**a**) X-ray diffraction patterns. (**b**) Differential scanning calorimetry curves of as-spun, 3-h, and 6-h hydrothermally treated Ti_50_Cu_32_Ni_15_Sn_3_ metallic glass, respectively.

Figure 1a,b show the X-ray diffraction (XRD) patterns and differential scanning calorimetry (DSC) traces of the as-spun, 3HRS, and 6HRS Ti_50_Cu_32_Ni_15_Sn_3_ alloys, respectively.

As seen in Figure 1a, all samples display a broad halo pattern, which is characteristic of an amorphous phase [19]. This indicates that the amorphous structure was retained even after the 3-h and 6-h hydrothermal treatments.

Figure 1b presents the DSC curves, revealing one endothermic reaction (T_g_, glass transition) followed by two exothermic reactions (T_x1_ and T_x2_, crystallization). As summarized in Table 1, the glass transition and crystallization onset temperatures (T_g_, T_x1_, and T_x2_), as well as the integral area of the crystallization peak (ΔH), remained nearly identical for the as-spun, 3HRS, and 6HRS alloys.

Collectively, the data presented in Figure 1a,b and Table 1 provide clear evidence that the as-spun Ti_50_Cu_32_Ni_15_Sn_3_ alloy is an amorphous phase and that this phase is successfully preserved throughout the hydrothermal process [19,20,21]. This retention of the amorphous phase implies that the samples maintain their inherent flexibility even after the formation of the oxide layer, which is crucial for their potential durability and long-term stability in photoelectrochemical devices and potentially in other flexible electronic and photoelectrochemical applications.

**Figure 2:**
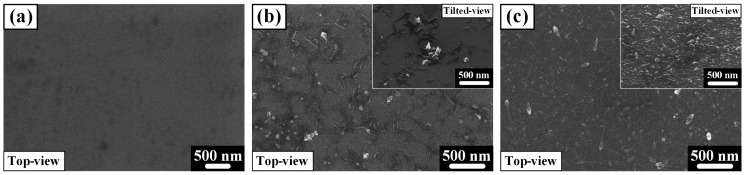
Surface morphology of the Ti_50_Cu_32_Ni_15_Sn_3_ alloy. Secondary electron micrographs of the (**a**) as-spun, (**b**) 3-h, and (**c**) 6-h hydrothermally treated Ti_50_Cu_32_Ni_15_Sn_3_ metallic glass.

Figure 2a–c present FE-SEM images of the oxide morphology on the as-spun, 3HRS, and 6HRS Ti_50_Cu_32_Ni_15_Sn_3_ alloys, respectively.

Figure 2a shows that the as-spun alloy has a smooth surface, devoid of any metal oxides. In contrast, the 3HRS sample in Figure 2b exhibits localized formation of oxides with a relatively uniform shape. This indicates that while oxides were formed by the hydrothermal process, the coverage was non-uniform due to the insufficient processing time of 3 h.

As shown in Figure 2c, the 6HRS sample displays a relatively uniform and continuous spread of oxides across the surface. A 45° tilted image (inset of Figure 2c) further reveals that these oxides grew randomly and overlapped with each other, forming a dense layer.

The collective results from Figure 2 demonstrate that longer hydrothermal processing time leads to a more uniform distribution of oxides on the surface of the Ti_50_Cu_32_Ni_15_Sn_3_ alloy. Based on a previous study with the same composition, temperature, and pressure conditions, the oxides in the layer are primarily the TiO_2_ anatase phase. It was also reported that Sn oxide is mixed with the TiO_2_ to create a synergistic effect, analogous to a SnO_x_-TiO_2_ junction or Sn-doped TiO_2_ [18]. Therefore, the oxides in the present study are also expected to be a mixture of Sn oxide and the TiO_2_ anatase phase.

The observed increase in oxide uniformity and layer density with longer hydrothermal duration, as qualitatively seen in FESEM images (Figure 2), is further corroborated by XPS depth profiling, which allows a quantitative assessment of the oxide layer thickness.

**Figure 3:**
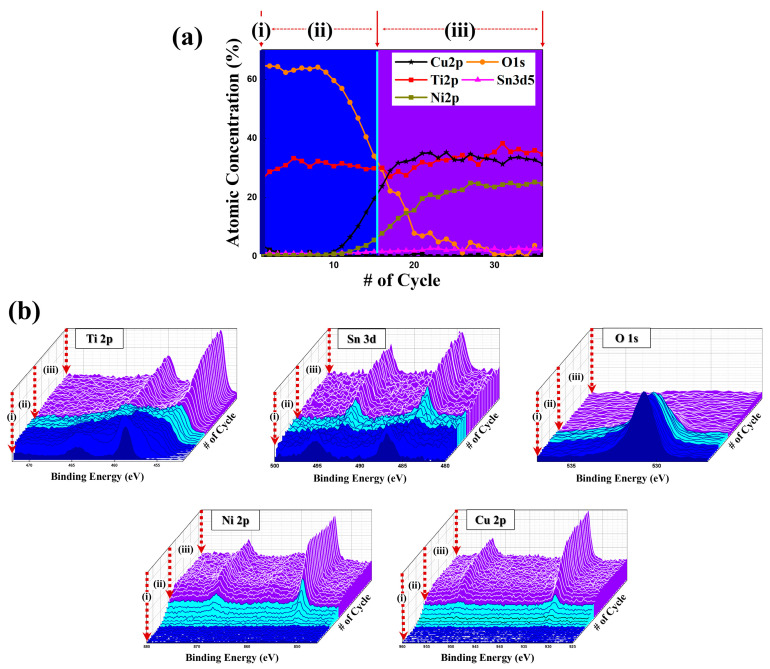
XPS depth profile analysis of the 6-h hydrothermally treated Ti_50_Cu_32_Ni_15_Sn_3_ alloy. (**a**) Atomic concentration depth profiles of Ti, Cu, Ni, Sn, and O within oxygen-rich surface layer (i), a gradual transition layer (ii), and a metallic substrate (iii). (**b**) Profile montage plots of each element.

Figure 3a,b show the XPS depth profiling analysis of the 6HRS sample, which exhibited the most uniform oxide layer. The binding energies in the XPS spectra were calibrated using the C 1s peak at 264.6 eV.

As illustrated in Figure 3a, the atomic concentration profile of the 6HRS sample from the surface can be divided into three distinct regions: (i) a surface layer, (ii) an oxide layer with oxygen permeation, and (iii) a metallic layer where oxygen is absent. In the surface layer (i), only O 1s, Ti 2p, and Sn 3d are present. Copper and nickel were not detected until a sufficient depth was reached in the oxide layer (ii). The oxygen concentration gradually decreases with depth, which corresponds to the point where the concentrations of Cu and Ni begin to increase. This trend is consistent with a previously reported 96-h hydrothermal process [18], confirming that a similar nanostructure can be achieved in a significantly shorter 6-h process. The oxide thickness of the 6HRS sample was estimated from the XPS depth profile to be ~27–40 nm, corresponding to the depth region where the O 1s atomic concentration reached its maximum. For comparison, our previous study on the same alloy composition showed that a much longer hydrothermal process of 96 h produced an oxide thickness of ~50 nm [18]. Taken together, these results indicate that the hydrothermal duration directly affects not only the density and uniformity of the oxide layer but also its thickness. This time dependence underscores the importance of systematically optimizing the duration in order to achieve oxide layers with superior structural continuity and photoelectrochemical performance.

Figure 3b shows the profile montage plots for each element. As with the atomic concentration data, Cu 2p and Ni 2p signals are absent from the surface layer (i) and increase with depth, a trend opposite to that of the O 1s signal [22,23,24,25].

**Figure 4:**
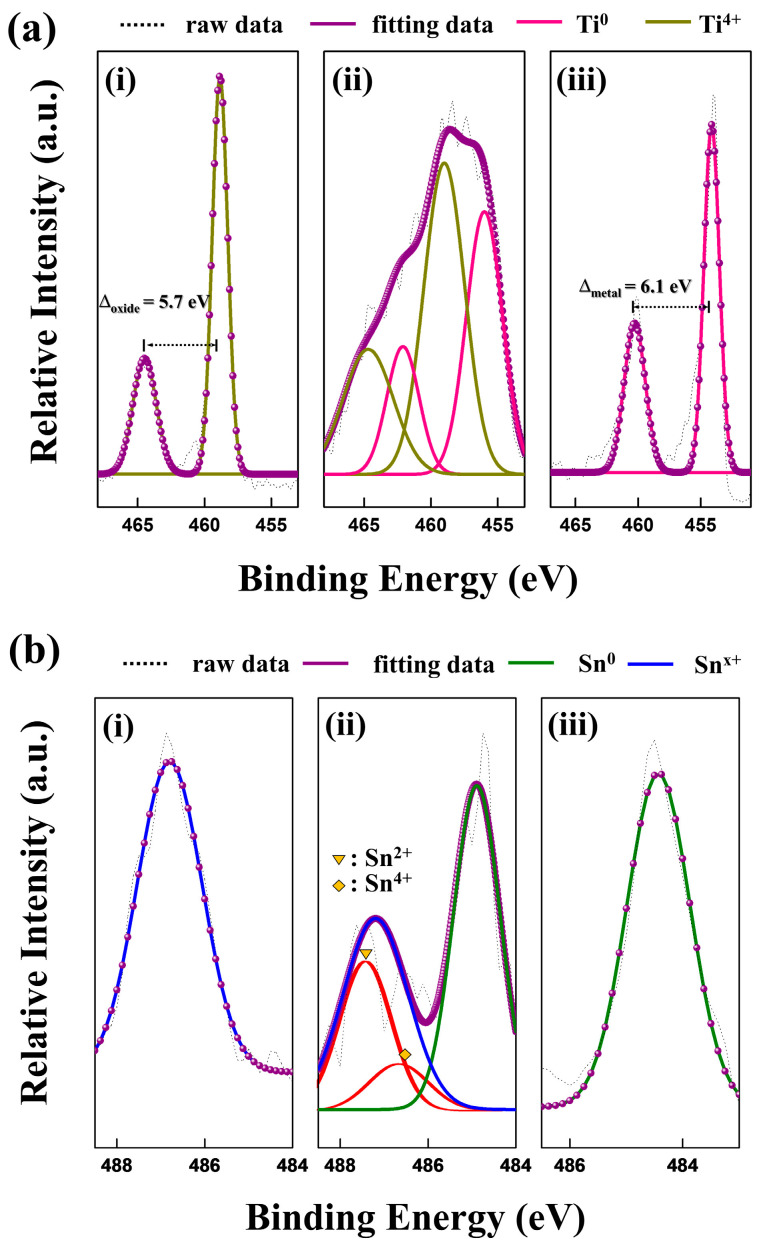
High-resolution XPS spectra of the 6-h hydrothermally treated Ti_50_Cu_32_Ni_15_Sn_3_ alloy. (**a**) The Ti 2p and (**b**) Sn 3d5/2 core-level spectra for distinct chemical states within the (i) surface layer, (ii) intermediate oxide layer, and (iii) metallic substrate. In ((**b**)-ii), the Sn 3d_5_/_2_ spectrum clearly resolves the contributions from Sn^2+^ and Sn^4+^ states, highlighted by the red lines, revealing the coexistence of mixed valence states in the intermediate oxide layer.

In the surface layer (i) and the metallic layer (iii), the Sn 3d and Ti 2p spectra each consist of a single doublet peak. However, multiple mixed peaks are observed within the intermediate oxide layer (ii). The specific chemical states of these elements are further detailed through binding energy deconvolution in Figure 4.

High-resolution XPS spectra for Ti 2p (a) and Sn 3d_5/2_ (b) are shown in Figure 4, depicting the chemical states within the surface (i), oxide layer (ii), and metal layer (iii) of the sample.

In the Ti 2p spectra (a) of the surface (i), the doublet at 464.5 eV (2p_1/2_) and 458.8 eV (2p_3/2_) arises from spin-orbit splitting. These peaks are consistent with the Ti4+ state in the TiO_2_ lattice [26]. In the oxide layer (ii), the TiO_2_ peaks were detected simultaneously with a doublet corresponding to metallic Ti (Ti^0^), with peaks at 464.7 eV (2p_1/2_) and 459.0 eV (2p_3/2_). As expected, the metal layer (iii) shows a doublet corresponding exclusively to the Ti0 state. Deconvolution of the Ti 2p binding energy thus indicates that TiO_2_ exists on the surface, while the oxide layer is a mixture of TiO_2_ and metallic Ti.

In the Sn 3d_5/2_ spectra (b) of the surface (i), the peak for Sn^x+^ is located at 486.8 eV. This Sn^x+^ peak represents a mixture of Sn^2+^ and Sn^4+^ states. According to the literature, the 3d_5/2_ peak for Sn^2+^ is located around 486.7 eV, and that for Sn^4+^ is around 486.5 eV; their close proximity makes them difficult to distinguish [27]. In Figure 4b(i), since the Sn^2+^ and Sn^4+^ peaks cannot be distinguished from each other, they are referred to as Sn^x+^ and indexed with a single peak corresponding to a SnO_x_ structure, a mixture of SnO and SnO_2_ lattices. Although this distinction is difficult, we can predict the phase transition as the process progresses [28], as the unstable Sn^2+^ state tends to gradually change to Sn^4+^ through the following reactions [29]:

4SnO(s) → Sn_3_O_4_(s) + Sn, (2)

Sn_3_O_4_ → 2SnO_2_ + Sn, (3)

Sn + O_2_ → SnO_2_(s), (4)

In the oxide layer (Figure 4b(ii)), a doublet is seen with peaks representing Sn^x+^ at 487.2 eV and metallic Sn^0^ at 484.9 eV. This indicates the coexistence of both SnO_x_ and metallic Sn within this layer [30]. The positive shift of the Sn 3d5/2 peak to 487.2 eV arises from the complex chemical environment and lattice effects in this multi-component system, which contains tin oxide, titanium oxide, and metallic species. Incorporation of multiple elements into the lattice induces local distortion, modifying M–O bond lengths and angles, and consequently reducing the electron density around oxygen atoms [31,32,33]. This electron deficiency increases the core-level binding energy of Sn and Ti, as observed in XPS, consistent with widely reported shifts in multi-component oxide systems [34,35].

The Sn 3d_5/2_ spectrum of the metal layer in Figure 4b(iii) shows a peak that can be assigned to the metallic Sn^0^ state. The locations of all deconvoluted peaks are listed in Table 2. Through XPS analysis of the 6HRS sample, we were able to confirm the presence of a mixed SnO_x_–TiO_2_ layer on the surface. The underlying oxide layer contains a mixture of metallic Ti, metallic Sn, SnO_x_, and TiO_2_. The selective oxidation of Ti and Sn, and the non-oxidation of Cu and Ni, can be explained by the standard heat of formation (ΔH_f_) for their respective oxides. Since the ΔH_f_ values for TiO_2_ anatase (−938.72 kJ/mol) and SnO_2_ (−577.6 kJ/mol) are significantly more negative than those for CuO (−314.8 kJ/mol) and NiO (−240.2 kJ/mol) [36,37], Ti and Sn are preferentially oxidized in the hydrothermal environment.

Photoelectrochemical analysis was performed to evaluate the water-splitting characteristics of the oxides formed on the amorphous alloy.

**Figure 5:**
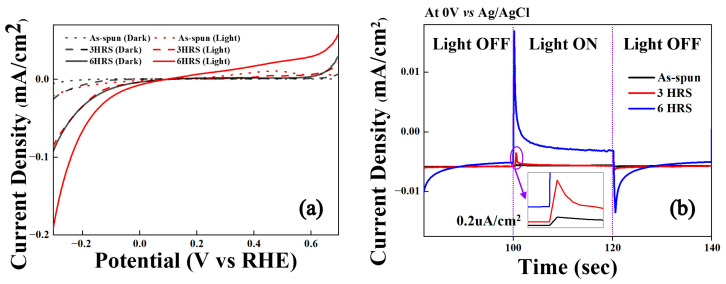
Photoelectrochemical (PEC) performance of the Ti_50_Cu_32_Ni_15_Sn_3_ alloys. (**a**) Linear sweep voltammetry (LSV) curves measured under dark and light illumination. (**b**) Chronoamperometry (CA) measurements conducted at 0 V versus Ag/AgCl with alternating illumination and dark periods.

Figure 5a illustrates the linear sweep voltammograms for the as-spun, 3HRS, and 6HRS ribbons. At −0.3 V vs. RHE, the photocurrent densities were −0.021 mA/cm^2^ for the as-spun ribbon, −0.024 mA/cm^2^ for the 3HRS sample, and a significantly higher −0.187 mA/cm^2^ for the 6HRS sample. Similarly, at +0.6 V vs. RHE, the photocurrent densities were 0.005, 0.006, and 0.03 mA/cm^2^ for the as-spun, 3HRS, and 6HRS samples, respectively. The current density of the alloy was consistently higher under light irradiation than in the dark and increased with longer hydrothermal processing time. For example, at −0.3 V vs. RHE, the 6HRS sample exhibited a current density approximately twice as high under illumination compared to dark conditions and about nine times higher than that of the as-spun ribbon. In addition to photocurrent density, the 6HRS sample also demonstrated the lowest onset potentials for both the Hydrogen Evolution Reaction (HER) and the Oxygen Evolution Reaction (OER).

Figure 5b presents the chronoamperometry curves for the Ti_50_Cu_32_Ni_15_Sn_3_ alloys, measured at 0.197 V vs. RHE under alternating 20-s light ON/OFF cycles. Overall, the current density increased under light irradiation. However, the enhancement was minor for the as-spun and 3HRS ribbons compared to the 6HRS sample. The increased photocurrent densities were 0.2, 0.35, and 2.8 μA/cm^2^ for the as-spun, 3HRS, and 6HRS samples, respectively, indicating that the 6HRS sample exhibited a photocurrent response roughly nine times greater than that of the 3HRS sample.

The superior performance of the 6HRS sample can be attributed to a dual-synergy effect. First, the highly uniform and continuous oxide layer, as confirmed by FE-SEM (Figure 2c), significantly increases the active surface area for light absorption, generating more photo-induced charge carriers. Second, the coexistence of TiO_2_ and SnO_x_ phases creates a heterojunction structure, which facilitates efficient charge separation and transport. This mixed-phase structure effectively suppresses electron-hole recombination, leading to a substantial increase in photocurrent density.

For a more comprehensive understanding of the effect of process duration, these results can be compared with our previous study on the same Ti_50_Cu_32_Ni_15_Sn_3_ alloy subjected to a much longer 96-h hydrothermal treatment [18]. In that study, the photocurrent density at −0.3 V vs. RHE reached approximately −11.1 mA/cm^2^, highlighting a significant difference in absolute performance between the two processes. The 96HRS sample also exhibited a substantially higher photocurrent and a sharper on/off response, indicating more efficient charge separation and faster carrier transfer. This superior response, along with its stable current under prolonged illumination, is attributed to a more mature and well-developed oxide layer resulting from the extended hydrothermal treatment. Importantly, the 6HRS sample was still able to show a clear and stable photoresponse, demonstrating that the underlying mechanisms for charge generation and transfer are successfully established even in the early stages of oxide growth. This highlights the importance of systematically optimizing hydrothermal duration to control the maturity of the oxide layer and its resulting PEC performance.

## 4. Conclusions

This study identified an optimal hydrothermal process time for the uniform formation of metal oxides on the surface of a Ti_50_Cu_32_Ni_15_Sn_3_ alloy. Hydrothermal treatments were conducted at 220 °C for 3 and 6 h using 150 mL of distilled water, with an as-spun ribbon serving as a control for comparison. The as-spun ribbon was confirmed to be in an amorphous phase, which was successfully retained even after both hydrothermal treatments. FE-SEM images of the oxide morphology showed that the as-spun ribbon had a smooth, oxide-free surface. The 3-h (3HRS) sample exhibited localized oxide formation with a regular shape, while the 6-h (6HRS) sample displayed a relatively uniform and continuous layer of randomly grown, overlapping oxides. XPS depth profiling analysis of the 6HRS sample revealed selective oxidation, where only Ti and Sn were oxidized, while Cu and Ni remained in their metallic state [22,23,24,25]. The analysis further confirmed that the surface layer consists of a mixed SnO_x_–TiO_2_ phase, and the underlying oxide layer is a mixture of metallic Ti and Sn, along with their respective oxides.

Furthermore, photoelectrochemical analysis showed that the 6HRS sample exhibited superior water-splitting properties compared to the other samples. This enhanced performance is attributed to the uniformly spread oxide layer, which is a mixed phase of TiO_2_ and SnO_x_. The coexistence of these oxides can be engineered to tune the band structure, thereby improving light absorption and carrier separation [18]. This study demonstrates that hydrothermal duration is a critical parameter for controlling the morphology and composition of the oxide layer, which directly impacts photoelectrochemical performance. While a previous study showed that a much longer 96-h treatment could achieve even higher photocatalytic activity, our results confirm that time control is the key to optimizing the oxide layer for superior photoresponse. Our time-controlled hydrothermal process using only water provides a viable and efficient strategy to simplify the manufacturing of high-efficiency photoelectrodes, with the potential to contribute to a wider range of electronic and photoelectrochemical applications of MGs. This work highlights the fundamental role of process control in tailoring functional surfaces on metallic glasses.

## Figures and Tables

**Table 1:** Thermal stability of the Ti_50_Cu_32_Ni_15_Sn_3_ alloys. The glass transition (T_g_) and crystallization onset (T_x_) temperatures, and the heat of crystallization (ΔH) for the as-spun, 3-h, and 6-h hydrothermally treated Ti_50_Cu_32_Ni_15_Sn_3_ metallic glass.

Sample	T_g_ (K)	T_x1_ (K)	ΔH_1_ (J/g)	T_x2_ (K)	ΔH_2_ (J/g)
**As-spun**	691.4	732.8	–36.7	767.7	–49.5
**3HRS**	692.5	729.5	–39.4	766.5	–53.6
**6HRS**	691.6	729.8	–37.5	766.9	–53.5

**Table 2:** Chemical states and binding energy values of 6-h hydrothermally treated Ti_50_Cu_32_Ni_15_Sn_3_ alloy. The deconvoluted binding energies and corresponding chemical states for the Ti 2p and Sn 3d_5/2_ core-level peaks, as analyzed by X-ray photoelectron spectroscopy on the 6-h hydrothermally treated Ti_50_Cu_32_Ni_15_Sn_3_ metallic glass.

6HRS	Binding Energy (eV)					
	Ti 2p			Sn 3d_5/2_		
Layer	Surface	Oxide	Metal	Surface	Oxide	Metal
	**Ti^4+^**	**Ti^4+^**		**Sn^x+^**	**Sn^x+^**	
	464.5	464.7		486.8	487.2	
	458.8	459.0				
		**Ti^0^**	**Ti^0^**		**Sn^0^**	**Sn^0^**
		462.1	461.4		484.9	484.4
		456.0	455.3			

## Data Availability

The original contributions presented in this study are included in the article. Further inquiries can be directed to the corresponding author.
